# Long-Term Results of Postoperative Hypofractionated Accelerated Breast and Lymph Node Radiotherapy (HypoAR) with Hypofractionated Boost

**DOI:** 10.3390/curroncol28050300

**Published:** 2021-09-07

**Authors:** Ioannis M. Koukourakis, Marianthi Panteliadou, Axiotis G. Giakzidis, Christos Nanos, Ioannis Abatzoglou, Alexandra Giatromanolaki, Michael I. Koukourakis

**Affiliations:** 1 1st Department of Radiology, Radiotherapy Unit, Aretaieion University Hospital, Medical School, National and Kapodistrian University of Athens, 11528 Athens, Greece; koukourioannis@gmail.com; 2 Department of Radiotherapy/Oncology, Democritus University of Thrace, University Hospital of Alexandroupolis, 68100 Alexandroupolis, Greece; marian_panteliadou@yahoo.gr (M.P.); radiotheralex@gmail.com (A.G.G.); c_nanos@hotmail.com (C.N.); abadzoglou@yahoo.gr (I.A.); 3 Department of Pathology, Democritus University of Thrace, University Hospital of Alexandroupolis, 68100 Alexandroupolis, Greece; agiatrom@med.duth.gr

**Keywords:** breast cancer, conservative surgery, radiotherapy, hypofractionation, acceleration

## Abstract

We report long-term results (median follow-up 12 years) of hypofractionated accelerated radiotherapy (HypoAR) in patients treated with breast-conserving surgery. In total, 367 women were treated with HypoAR. Axillary and supraclavicular area (ASA) were treated in patients with involved nodes. In total, 290 patients (scheme A) received 3.5 Gy/day ×10 fractions (breast/ASA) followed by two 4 Gy fractions with electrons to the affected breast quadrant within 16 days. In total, 77 patients (Scheme B) received 2.7 Gy/day for 16 consecutive fractions (breast/ASA) within 22 days, while concurrently, the affected breast quadrant received an electron booster dose of 0.8 Gy for the first 13 fractions. Amifostine was offered to 252/367 patients. Early radiation toxicity was minimal. Regarding late toxicities, symptomatic breast edema was noted in 2.2%, asymptomatic breast fibrosis in 1.9%, and arm lymphedema in 3.7% of patients. Amifostine reduced early radiation dermatitis (*p* = 0.001). In total, 2.2% of patients developed contralateral breast and 1.6% other carcinomas. Locoregional recurrence (LR) occurred in 3.1% of patients (0% for in situ carcinomas). Positive margins after surgery, extracapsular node invasion, and HER2-enriched/triple-negative tumors were linked with significantly worse LR-free survival. The involvement of more than three nodes and luminal type other than A were independent prognostic variables of metastasis and death events. HypoAR delivering a biological dose of 50–52 Gy to the breast/ASA is a safe and effective therapy for patients treated with conservative surgery. The risk of carcinogenesis is low. Positive surgical margins, extracapsular node invasion, and HER2-enriched/triple-negative phenotypes appear as a cluster of features linked with a higher risk for locoregional relapse.

## 1. Introduction

Conservation of the breast with partial mastectomy techniques followed by postoperative radiotherapy has been an established organ-preserving therapy during the last three decades [1]. Radiotherapy minimizes the risk of locoregional recurrence and decreases death rates in high-risk patients [2]. Conventional radiotherapy (CRT) delivering 50 Gy to the breast and/or axilla and a 16 Gy boost to the tumor bed demand a 7-week schedule that is inconvenient to patients and also results in the overloading of waiting lists in many cancer centers. Due to this inconvenience, patients may decline radiotherapy, especially when residing away from radiotherapy centers [3].

Condensing the radiotherapy schedule using hypofractionation has long been considered prohibited, after the ‘lessons from complications’ paper published by Fletcher GH in 1991, one of the founders of modern radiotherapy [4]. Developments in clinical radiobiology, however, strongly questioned the barriers set (omission of a sentence). An early study by Baillet et al., published in 1990, comparing CRT to a 4-fraction radiotherapy scheme, strongly supported the feasibility of HypoAR in breast cancer [5]. Our ‘mind constructs’ regarding fractionation started to drastically shift after the analysis of large clinical data of prostate cancer, suggesting that tumors may have low α/β-ratio values [6]. In 2005, Yarnold et al., analyzing the long-term results of a randomized trial on 1410 breast cancer patients, estimated that the α/β-ratio of breast cancer is similar to the normal breast tissues, with a median value of 3.6 Gy [7]. This was subsequently confirmed in a radiobiological analysis of the START A randomized trial, providing a median α/β-ratio of 4.6 Gy for breast cancer tissue [8]. Today, the Canadian trial delivering 42,5 Gy in 16 fractions has been worldwide established as an RT schedule for routine use in breast cancer patients treated with conservative surgery [9].

Our early experience, published in 2002, treating breast cancer patients with a 3.5 Gy per fraction for 10 consecutive fractions followed by booster radiotherapy dose provided encouraging results [10]. Indeed, interim reports of subsequent studies with the same schedule applied after partial mastectomy [11] or modified radical mastectomy [12] confirmed the excellent tolerance and efficacy of the regimen. Here, we report long-term results (10–17 years of follow-up) from our one-center trial with HypoAR in patients treated with breast-conserving surgery. Analysis of risk factors for local and distant relapse, as well as for secondary tumors, is also provided.

## 2. Materials and Methods

From January 2003 to December 2010, 367 women with breast cancer treated with breast-conservative surgery were recruited in a prospective trial of hypofractionated and accelerated radiotherapy (HypoAR), focusing on tolerance and efficacy. The study was approved by the Ethics and Research Committee of the University Hospital of Alexandoupolis (SD24, date 6 January 2004). Preliminary results of the trial were published in 2009 [11]. The current study analyzes mature results after 10 to 17 years of follow-up (median 12 years). A follow-up exceeding 10 years was available for 330/367 patients. For 10/367 patients, the follow-up ranged from 2–5 years, while for 27/367 patients, this was shorter than 2 years. In these latter two groups of patients, all patients had no evidence of local or distant disease at the time of the last examination.

All patients had a performance status of 0 (WHO scale). Patients previously treated with radiotherapy in the chest area, pregnant women, patients with concurrent hematological or other malignancies, and patients with significant heart, lung, liver, renal, and psychiatric disease were excluded. All patients gave written informed consent. Table 1 shows the patient, disease, and medical treatment characteristics. Partial mastectomy and axillary node dissection (limited or extensive) were performed in 336/367 patients, while in 31/367 women, axillary dissection was not performed under a clinical/radiological N0,1 stage. Neo-adjuvant chemotherapy was performed in only 12/367 patients.

### 2.1. Radiotherapy Schemes

Radiotherapy was delivered with 3-D-conformal techniques using a 6–18 MV linear accelerator (ELEKTA), endowed with a multileaf collimator. Following CT simulation, treatment planning was performed with the ‘Plato’ (Nucletron) planning system. The breast was treated by applying two to four tangential X-ray fields. All patients with involved nodes received radiotherapy to the axillary and supraclavicular area with anteroposterior fields. None of the patients received internal mammary area irradiation. All patients received a boosted dose to the affected quadrant. Two different radiotherapy schemes were applied, according to the physician’s discretion, as follows:

Scheme A (290 patients): Breast and axillary area (when included) received 3.5 Gy per day for 10 consecutive fractions within 12 days. Subsequently, the quadrant where the primary tumor was located received two additional fractions of 4 Gy with 10–15 MeV electrons (with appropriate adjustment with bolus according to the anatomy). The overall treatment time was 16 days.

Scheme B (77 patients): Breast and axillary area (when included) received 2.7 Gy per day for 16 consecutive fractions within 22 days. The affected breast quadrant received a concomitant booster dose of 0.8 Gy (3.5 Gy/day to the quadrant) for the first 8 fractions. The overall treatment time was 22 days.

Schedule B was formulated to simulate the Canadian schedule with concomitant booster dose to the affected quadrant. The choice between schedule A and B was at the discretion of the physician. Schedule B was more cumbersome, and it was soon abandoned.

The radiobiological dose analysis is reported in Table 2. The normalized total dose or otherwise named EQD2 (equivalent dose to a 2 Gy/fraction scheme), corrected for overall treatment time, was calculated using a previously proposed formula [13], EQD2_(T)_ = D [(α/β + d)/(α/β + 2)] + λ (Tc − To), where ‘Tc’ is the number of days required for the delivery of the EQD2 using a conventionally fractionated scheme, ‘To’ is the number of days required for the delivery of the current scheme, and ‘λ’ is the estimated daily dose consumed to compensate for rapid tumor repopulation. For cancer and normal breast area tissues, an α/β ratio of 4 Gy was considered as calculated by Yarnold et al. [7,8]. For cancer cells, a λ-value of 0.4 Gy was considered. For normal tissue late effects, a λ-value of 0.2 Gy was adopted in radiobiological calculations [14] (Appendix A).

Amifostine, delivered subcutaneously, was offered to 252/367 patients, according to their consent to receive cytoprotection, following discussion on the eventual benefits and side effects expected from the drug, as previously reported [10].

### 2.2. Toxicity Evaluation

The NCI (National Cancer Institute, Bethesda, MD, USA) Common Terminology Criteria for Adverse Events Version 5 scale was used to assess chemotherapy and acute radiation toxicity [15]. The LENT-SOMA (late effects of normal tissue subjective, objective, management, and analytic scales) scale was used for the clinical assessment of late sequel [16]. For simplicity, certain modifications were adopted, as shown in Table 3 and Table 4.

### 2.3. Statistical Analysis

The GraphPad Prism 7.00 version package (San Diego, CA, USA) was used to perform statistical analysis and graph presentation. The SPSS program was used to perform multivariate analysis. The Fisher’s exact test was used to compare categorical variables and the unpaired two-tailed *t*-test for group analysis of continuous variables. Survival curves were plotted using the Kaplan–Meier method, and the log-rank test was used to determine statistical differences between life tables. A Cox proportional hazard model, including variables significant at univariate analysis, was used for multivariate analysis of locoregional relapse, metastasis, and death events. A *p*-value of < 0.05 was considered for significance.

## 3. Results

### 3.1. Radiation Toxicity

Consistent with the preliminary reports [11], early toxicity was minimal (Table 3). Brisk erythema with/or without patchy moist skin desquamation was noted in 46/367 (12.5%), while moderate breast edema in 10/367 (2.7%) patients. Mild discomfort of the irradiated area was reported by 34 (9.2%) and moderate pain that did not require analgesics in 10 (2.7%) patients. There was no case of clinical or radiographically detected acute pneumonitis. The administration of amifostine was significantly related to a reduced toxicity score of radiation dermatitis (*p* = 0.001) and breast edema (*p* = 0.03) but had no effect on the score of pain. Early toxicities were similar between patients receiving the schedule A and B. There was a trend for schedule B to produce higher rates of acute dermatitis (*p* = 0.06).

Late toxicities were also low (Table 4). Of interest, all toxicities were recorded within the first 24 months of follow-up, and there were no new or deteriorating toxicities after that. Symptomatic tolerable breast edema was noted in 8/367 (2.2%), while definite asymptomatic fibrosis of the breast in 7 (1.9%) patients. Skin telangiectasias were noted only within the tumor bed in 77 (21%) patients (dense in 3.2%). Evident deterioration of post-surgical arm lymphedema was recorded in 14 (3.7%) patients. Chest CT scan confirmed limited signs of in-field lung fibrosis in 37 (10.1%) patients, and there was no case of symptomatic lung disease. The administration of amifostine had a marginal effect on the appearance of fibrosis (*p* = 0.09) and of breast edema (*p* = 0.06). There was no difference between schedule A and B.

### 3.2. Second Carcinomas

Within up to 17 years of follow-up, 8 (2.2%) patients developed a new primary cancer of the contralateral breast. Six out of 367 (1.6%) patients developed non-breast carcinomas (2 ovarian, 1 lung, 1 gastric, 1 skin outside the radiation portals, and 1 vulvar cancer).

### 3.3. Control of In Situ Carcinoma

Out of 12 patients with in situ carcinoma treated with HypoAR (6 patients with the regimen A and 6 with the regimen B), none of them presented with locoregional recurrence, and all patients were alive with no evidence of disease within 120–180 months of follow-up.

### 3.4. Locoregional Control

**Figure 1:**
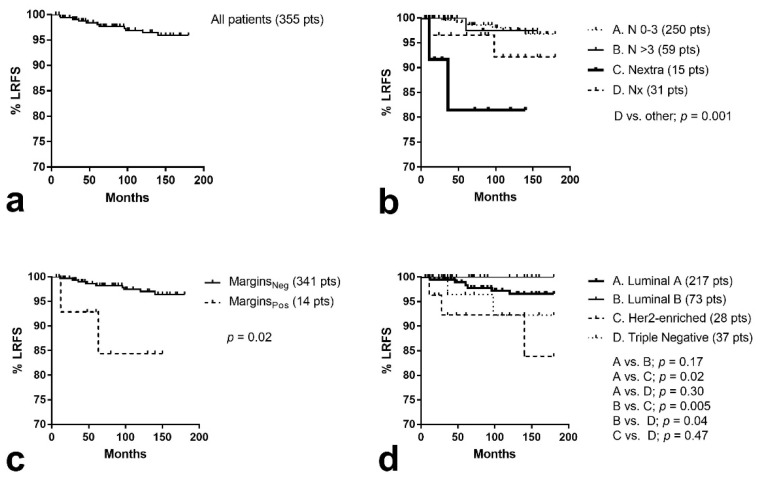
Kaplan–Meier survival curves of local relapse-free survival (LRFS): (**a**) all patients included, (**b**) stratified for nodal involvement (extra = extracapsular, x = unknown), (**c**) according to pathological status of resection margins, (**d**) stratified for luminal molecular breast cancer subtypes.

Locoregional recurrence (LR) occurred in 11/355 (3.1%) patients (in situ cancers excluded from analysis), as shown in Figure 1a. Excluding the 27 patients lost to follow-up at an interval of fewer than two years, the locoregional recurrence rate was 3.3%. Excluding patients with positive surgical margins, this was 2.6% (9/341).

The Kaplan–Meier locoregional recurrence-free survival (LRFS) curves and relevant univariate analysis are shown in Figure 1 and Table 5. Positive margins after surgery were linked with significantly worse LRFS (*p* = 0.02; Figure 1c). Locoregional recurrence increased to 14.2% (2/14) in these patients. Extracapsular node invasion was also significantly linked with worse LRFS (*p* = 0.01; Figure 1b), with 13.3% (2/15) recurrence rates. Although the omission of lymphadenectomy had a slightly higher LR rate (7.9%), this was not statistically significant (*p* = 0.26). Moreover, extensive lymphadenectomy (>16 dissected nodes) did not have any effect on LRFS (*p* = 0.97).

The group of patients with HER2-enriched or triple-negative tumors also had a higher risk of locoregional recurrence (*p* = 0.009; Figure 1d). The LR rate was 7.7% (5/65). T-stage, lymphovascular space invasion, and multifocality were not linked with increased risk of LR. Comparing the lobular to the NOS or other histology subtypes, there was no significant difference. 

The type of HypoAR regimen and the administration of amifostine had no effect on LRFS.

In a multivariate analysis model including all parameters that were significant at univariate analysis, positive margins, extracapsular nodal invasion, and HER-enrichment/triple-negative disease were independent prognostic variables of LR (Table 5).

### 3.5. Distant Metastasis and Overall Survival

At the time of analysis, 55/355 (15.5%) patients had been recorded with metastasis to distant organs (‘in situ’ cancers excluded from analysis). Kaplan–Meier univariate analysis showed that T1 stage, number of involved nodes less than four, and luminal type A were significantly related with better metastasis-free survival (*p* = 0.02, *p* = 0.001, and 0.0001, respectively).

**Figure 2:**
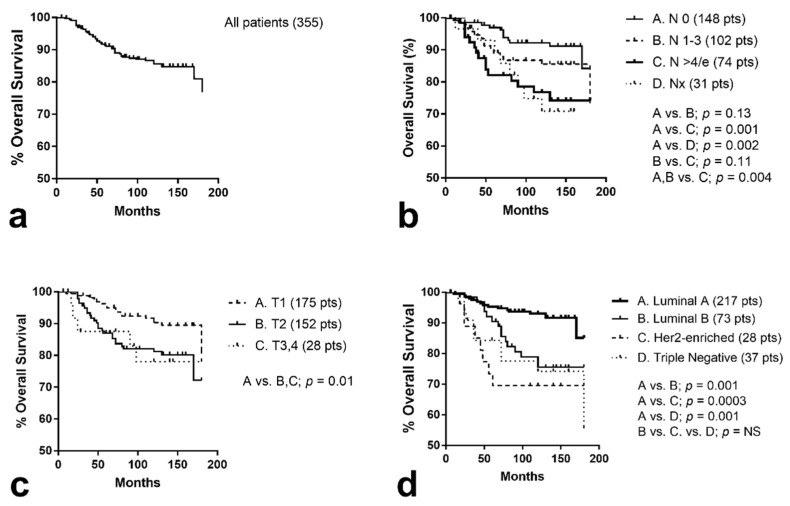
Kaplan–Meier survival curves of disease-specific overall survival (LRFS): (**a**) all patients included, (**b**) stratified for nodal involvement (e = extracapsular), (**c**) according to T stage, (**d**) stratified for luminal molecular breast cancer subtypes.

At the time of analysis, 68/355 had died, 20/68 from reasons not related to their breast cancer and 48/68 from breast cancer progression. Kaplan–Meier univariate analysis of disease-specific events showed that T1 stage, number of involved nodes less than 4 (Nx patients excluded), and luminal type A were significantly related with better distant metastasis-free survival (*p* = 0.01, *p* = 0.004 and <0.0001, respectively), as shown in Figure 2a–d.

In multivariate analysis of variables significant at univariate survival analysis, the involvement of more than three nodes and luminal type other than A were independent prognostic variables of metastasis and death events (Table 5).

## 4. Discussion

The usage of HypoAR after breast-conserving surgery for breast cancer patients is gradually increasing worldwide [17]. The confirmation of an α/β-ratio value of around 4 Gy for breast cancer tissues [7], which is equal to the one normal breast late responding tissues, encouraged the conduct of large randomized trials to evaluate the feasibility of the reduction of the number of visits of patients to the radiotherapy departments, obtained with simple hypofractionation with or without shrinkage of the overall treatment time. 

The recently published 10-year follow-up of the ‘FAST trial’ on breast-only irradiation confirmed the safety and efficacy of a close to ultra-hypofractionation, five-fraction (one fraction per week) regimen delivering 5.7 Gy/week [18]. This regimen delivers 28.5 Gy, thus an EQD2 of 46.07 Gy in 29 days with minimal 2-day acceleration. The Canadian trial on breast-only irradiation also proved that an accelerated 22-day regimen (42.5 Gy in 16 fractions) provides high efficacy, less acute toxicity, and improved quality of life compared to CRT [19]. This regimen delivers an EQD2 of 47.2 Gy in 22 days, thus with an acceleration of 10 days, which gives an EQD2-T for the breast (λ = 0.2 Gy) of 49.2 Gy. Similar results in terms of effectiveness and breast toxicity have been reported in the UK START A trial, where breast received 13 fractions of 3 Gy [20]. This regimen delivers an EQD2 of 45.5 Gy in 17 days. This regimen provides an acceleration of 14 days, so that the ΕQD2-T is estimated to be 48.3 Gy. All these schedules produce a similar toxicity to the conventionally fractionated regimen delivering 50 Gy to the breast. Our schedules A and B deliver a similar time-corrected biological dose of 47.35 and 50.24 Gy, respectively, to the whole breast and as expected late breast toxicity was minimal. Despite the slightly higher dose of schedule B, schedule A and B had similar tolerance and efficacy. As schedule B was more cumbersome (demanding daily administration of the booster field), it was soon abandoned by physicians. The very low early breast toxicity recorded was also an expected finding, as early responding tissues, like skin, have a higher α/β-ratio (above 10 Gy), which results in breast exposure to a significantly lower biological dose (estimated to 39 Gy) compared to CRT (50 Gy).

The administration of a booster dose to the affected breast quadrant is a policy not always followed or, at best, adopted in patients with high-risk of local relapse, like patients with positive surgical margins, large tumors at diagnosis, high-grade tumors, or even tumors with adverse molecular features [21]. This booster dose increases the local control of the disease but seems to not improve the overall survival of patients [22], and this is the reason why it is omitted in many cancer centers. The recommended dose is 10–16 Gy in 5–8 fractions or 12 Gy in 4 fractions [23]. Our trial design included two fractions of 4 Gy electron irradiation (equivalent to 12 Gy taking into account acceleration). This dose did not increase early toxicity. Regarding late toxicity, dense atelectasia within the booster field was noted in 3.2%, while palpable localized non-symptomatic fibrosis was recorded in 13% of patients. Given the very low toxicity and locoregional relapse rates observed in our trial and the estimated high death rates after local recurrence (as high as 32% and 59% in stage I and II disease, respectively [24]), we strongly recommend the delivery of a booster dose to breast cancer patients, especially to those with high-risk features.

Another issue that remains to be resolved in the breast-HypoAR practice is the fractionation applied for the treatment of axillary and supraclavicular areas. The Standardisation of Breast Radiotherapy UK START A and B trials showed that arm edema and shoulder stiffness had no different fraction sensitivity than breast and chest [25]. Nevertheless, higher arm/shoulder toxicity was noted in the cohort of patients receiving 13 fractions of 3.3 Gy, which gives a dose of 54 Gy (for α/β-ratio equal to 3 Gy) to the axilla, which, however, is higher than the 50 Gy delivered with CRT. Our patients with positive or unknown node status received ten fractions of 3.5 Gy in this area, without further boost, equivalent to a time-corrected 50–52 Gy of CRT. Conspicuous deterioration of the postoperative arm lymphedema above 4 cm was noted in 2.2%, and mild pain in 3.7% of patients. This confirms that, indeed, axillary tissues have a similar fractional radiosensitivity to breast tissues. 

We further analyzed the therapy features, and histological and molecular variables that affect prognosis. The two HypoAR schedules applied were equivalent in terms of local and metastasis-free survival rates and, moreover, amifostine did not have any effect on the efficacy of radiotherapy. Positive surgical margins and extracapsular nodal involvement were independent variables related to locoregional recurrence. Of interest, HER2-enriched and triple-negative tumors were related to increased locoregional relapse rates. These ominous prognostic features have also been identified in previous studies [26]. The tumor size, the number of involved nodes, or even the number of excised nodes did not have any effect. Whether increasing the dose in the affected quadrant or even the dose to the axilla for patients with limited surgical lymphadenectomy would improve locoregional control in patients with the above-mentioned characteristics is a sound hypothesis, even if toxicities may increase. Regarding the disease-specific survival, advanced T-stage, a high number of involved nodes, and luminal type other than A were independent variables of metastasis and prognosis.

Another issue that has been raised during the early era of breast cancer HypoAR studies is that long-term follow-up should also focus on an eventual higher carcinogenic risk of large radiotherapy fractions. Indeed, in an analysis by Kirova et al., radiotherapy for breast cancer has been associated with increased risk for the development of secondary carcinomas, especially of the lung, and sarcomas [27]. Authors analyzed 16,705 patients (13,472 treated with postoperative RT vs. 3233 treated with surgery alone) with a median follow-up of 10.5 years. The incidence of second malignancies was 4.42% (596/13,472) in patients receiving radiotherapy vs. 3.5% (113/3233) in patients receiving surgery alone [27]. In the Kirova series of patients, 35 developed sarcomas, 27 of them considered to be radiation induced (incidence 0.2%, 7.4-fold increased incidence compared to the non-RT group). The incidence of lung cancer was 0.4% (54/13.472) in the RT group vs. 0.1% (4/3.233) in the non-RT group. In our study, within a median follow-up of 12 years, the incidence of lung cancer was 0.3%, and we recorded no case of sarcoma. Kirova et al. reported that 52/58 women who developed secondary lung cancer had a smoking history, but we have no such data available to report for our series. The overall incidence of neoplasia was 1.6%, which is lower than the value recorded by Kirova et al., even in the non-RT group of patients. In contrast to the worries of enhanced carcinogenesis by hypofractionation, Schneider et al. reported biological models that suggest a reduced risk, which is in accordance with our findings [28]. Regarding the risk of contralateral breast cancer, Kirova et al. found no increase in the group of patients receiving RT. The risk was 8.2% (1.113/13,472) in the RT group vs. 7.1% (230/3234) in the non-RT group of patients. In our series, the risk of contralateral breast cancer was as low as 2.2%. Whether the administration of the anti-mutagenic agent amifostine in two-thirds of our patients also contributed to these effects deserves further investigation [29].

## 5. Conclusions

It is concluded that HypoAR is a safe and effective therapy for patients treated with conservative surgery. A time-corrected EQD2 of 48–50 Gy proved optimal for both breast and axilla/supraclavicular (omission of sentence). The risk of carcinogenesis is at least as low as the one expected from conventional radiotherapy. Positive surgical margins, extracapsular node invasion, and HER2-enriched/triple-negative phenotype appear as a cluster of characteristics implying a high risk for locoregional relapse. Although the use of booster radiation to the affected breast quadrant is not a standard practice in many institutions, the current study provides evidence that this can be safely applied in subgroups of patients with high risk, but its therapeutic value needs evaluation in randomized trials.

## Figures and Tables

**Table 1:** Patient, disease, and medical treatment characteristics, stratified also for RT schedule.

	All Cases	Schedule A	Schedule B	*p*-Value
	367	290	77	
**Age**				
Median	56	56	60	0.74
Range	26–84	26–84	29–81	
**Performance status**				
0	367	**290**	**77**	
**Histology**				
NOS (*)	338	264	74	0.41
Lobular	23	21	2	
Myeloid	4	3	1	
Mucinous	2	2	0	
**T-stage**				
Tis	12	6	6	0.22
T1	175	147	28	0.01 ^(#)^
T2	153	115	38	
T3	19	16	3	
T4	8	6	2	
**Multifocality**				
No	342	272	70	0.37
Yes	25	18	7	
**Lymphovascular space invasion**				
No	337	266	71	0.89
Yes	30	24	6	
**Resection Margins**				
Negative	335	279	74	0.96
Positive	14	11	3	
**Node involvement**				
0	160	124	36	0.45
1–3	120	79	23	
4–10	43	32	11	
>10	16	14	2	
extracapsular invasion	15	14	1	
unknown (**)	31	27	4	
**Grade (invasive NOS)**				
1	57	48	9	0.27
2	113	85	29	
3	156	126	30	
**ER status**				
Negative	75	65	10	0.07
Positive	292	225	67	
**PgR status**				
Negative	87	74	13	0.11
Positive	280	216	64	
**HER-2status**				
Negative	263	202	56	0.60
Positive	104	88	21	
**Luminal status**				
Luminal A (***)	225	169	56	0.51
Luminal B (****)	76	61	15	0.02 ^(##)^
Her-2 enriched	28	23	5	
Triple negative	38	37	1	
**Surgery (invasive tumors)**				
Conservative	335	284	71	0.30 ^(###)^
no axillary intervention	31	27	4	
axillary sampling/dissection	324	257	67	
dissected nodes 0–10	111	87	24	
dissected nodes 11–16	140	115	25	
dissected nodes >16	73	55	18	
Chemotherapy (*****)				
Hormonal therapy only	91	72	19	0.94
Neo-adjuvant	12	9	3	
Postoperative	264	209	55	

(*) NOS = not otherwise specified ductal cancer; (**) Treated with lumpectomy only; (***) Luminal A = ER positive/Her-2 negative. (****) Luminal B = ER positive/Her-2 positive; (*****) Trastuzumab was administered to all patients receiving chemotherapy for HER2-positive tumors; ^(#)^ Tis tumors were significantly more frequent in the schedule B (*p* = 0.01), but there was no difference in the distribution of T-stages in the two schedules (*p* = 0.22); ^(##)^ Triple-negative patients were significantly more frequently treated with schedule A (*p* = 0.02). No other difference between schedules was noted (0.51). ^(###)^ *p*-value refers to the comparison between patients receiving or not axillary surgery.

**Table 2:** Radiobiological analysis of the two HypoAR schemes.

	No Pts	Gy/f × f	PhysD (Gy)	EQD2n,c (Gy)	Time (Days)	Δt (Days)	EQD2n-T (Gy)	EQD2c-T (Gy)
**Regimen A:**	**290**							
Breast/Axilla (*)		3.5 × 10	35	43.75	12	18	47.35	50.90
Tumor quadrant		4 × 2	8	10.66	2	3	11.26	11.86
Total Tumor quadrant		3.5 × 10 + 4 × 2		54.41	16	21	58.61	62.76
**Regimen B:**	**77**							
Breast/Axilla (*)		2.7 × 16	43.2	48.24	22	10	50.24	52.24
Total Tumor quadrant		3.5 × 8 + 2.7 × 8		35 + 24.1 (total 59.1)	10 + 12 (total 22)	18	62.7	66.3

EQD2n,c: Equivalent Total Dose that would be delivered with 2 Gy per fraction, to normal and cancer tissues calculated for α/β = 4 Gy; f: Fraction; Δt: Days of acceleration of radiotherapy; EQD2n-T: EQD2 corrected for time, delivered to normal tissues (calculated λ = 0.2 Gy); EQD2c-T: EQD2 corrected for time, delivered to cancer (calculated for λ = 0.4 Gy); (*): Axilla was irradiated in patients with even one positive node or without axillary dissection.

**Table 3:** Early toxicity assessed with the NCI (National Cancer Institute, Common Toxicity Criteria Version 5)-based scale (findings with 3 months following the onset of radiotherapy).

	All Patients	Schedule A	Schedule B	*p*-Value
	367	290	77	
**Radiation dermatitis**				
0/1. None to Faint erythema	321 (87.5)	259 (89.3)	62 ((80.5)	0.06
2. Brisk erythema/Patchy moist desquamation	46 (12.5)	31 (10.7)	15 (19.5)	
3. Confluent moist desquamation	0 (0)	0 (0)	0 (0)	
4. Skin necrosis	0 (0)	0 (0)	0 (0)	
**Breast Edema (modified by authors)**				
0. None	296 (80.6)	237 (81.7)	59 (76.7)	0.58
1. Barely palpable/asymptomatic	56 (15.3	43 (14.8)	13 (16.8)	
2. Moderate/tolerable	10 (2.7)	7 (2.4)	3 (3.9)	
3. Requiring therapy	5 (1.4)	3 (1.0)	2 (2.6)	
**Pain (Breast/arm)**				
0. NoNone	330 (89.9)	263 (90.7)	67 (87.0)	0.31
1. Mild discomfort	34 (9.2)	24 (8.3)	10 (13.0)	
2. Moderate	3 (1.0)	3 (1.0)	0 (0)	
3. Requiring analgesics	0 (0)	0 (0)	0 (0)	
**Pneumonitis**				
0. None	367 (100)	290 (100)	77 (100)	
Other	0 (0)	0 (0)	0 (0)	

**Table 4:** Late toxicity assessed with the LENT-SOMA grading system (with modifications).

	All Patients	Schedule A	Schedule B	*p*-Value
	367	**290**	**77**	
**Breast edema**				
0. None	311 (84.7)	244 (84.1)	67 (87.0)	
1. Barely evident/Asymptomatic	48 (13.1)	41 (14.2)	7 (9.0)	
0. Evident/Tolerable	8 (2.2)	5 (1.7)	3 (4.0)	
1. Dysfunctional	0 (0)	0 (0)	0 (0)	
**Fibrosis/breast shrinkage**				
0. None	310 (84.5)	251 (86.6)	61 (79.2)	0.27
1. Palpable/evident in the booster field	50 (13.6)	34 (11.7)	14 (18.2)	
0. Marked breast shrinkage	7 (1.9)	5 (1.7)	2 (2.6)	
1. Very marked firmness/fixation	0 (0)	0 (0)	0 (0)	
**Skin telangiectasia (modified by authors)**				
0. None	290 (79)	231 (79.7)	59 (76.6)	0.83
1. Sparse (in the tumor bed)	65 (17.8)	50 (17.2)	15 (19.5)	
2. Dense (in the tumor bed)	12 (3.2)	9 (3.1)	3 (3.9)	
3. Outside the tumor bed	0 (0)	0 (0)	0 (0)	
**Skin atrophy or ulceration**				
0. None	367 (100)	290 (100)	77 (100)	0.20
**Arm lymphedema (compared to postoperative)**				
0. None	331 (90.2)	260 (89.7)	71 (92.2)	
1. 2–4 cm	28 (7.6)	25 (8.6)	3 (3.9)	
0. 4–6 cm	8 (2.2)	5 (1.7)	3 (3.9)	
**Pain (Breast/arm)**				
0. None	353 (96.2)	278 (95.8)	75(97.4)	0.76
1. Mild	13 (3.5)	11 (3.8)	2 (2.6)	
2. Moderate	1 (0.2)	1 (0.4)	0 (0)	
3. Severe	0 (0)	0 (0)	0 (0)	
**Lung fibrosis**				
0. None	330 (89.9)	260 (89.7)	70 (90.9)	0.75
1. Radiographic changes/asymptomatic	37 (10.1)	28 (9.6)	7 (9.1)	
2. Dense Radiographic changes/symptomatic	0 (0)	2 (0.7)	0 (0)	

**Table 5:** Local progression-free survival, distant metastasis-free survival, and disease-specific overall survival analysis.

	Univariate		Multivariate	
Variable	HR Ratio	*p*-Value	HR Ratio	*p*-Value
**Local Progression**				
T-stage (T3,4 vs. T1,2)	2.9	0.38		
N (extracapsular vs. other)	8.0	0.001	6.3	0.03
Dissected nodes (≥16 vs. <16)	1.0	0.97		
Margins (pos vs. neg)	5.1	0.02	6.7	0.01
Multifocality (yes vs. no)	2.9	0.33		
LVI (yes vs. no)	1.2	0.86		
Luminal (other vs. A, B)	5.0	0.009	3.4	0.05
Age (≤50 vs. >50)	1.5	0.46		
RT-scheme (A vs. B)	1.2	0.76		
Amifostine (yes vs. no)	1.2	0.74		
**Distant metastasis**				
T-stage (T2,3,4 vs. T1)	1.8	0.02	1.6	0.09
N (other vs. 0–3)	3.2	0.001	1.9	0.02
Dissected nodes (≥16 vs. <16)	1.1	0.79		
Margins (pos vs. neg)	2.3	0.21		
Multifocality (yes vs. no)	2.1	0.26		
LVI (yes vs. no)	1.9	0.19		
Luminal (other vs. A, B)	3.4	0.0001	3.1	<0.0001
Age (≤50 vs. >50)	0.6	0.07		
RT-scheme (A vs. B)	1.2	0.43		
Amifostine (yes vs. no)	1.2	0.44		
**Overall Survival**				
T-stage (T2,3,4 vs. T1)	2.1	0.01	2.0	0.07
N (other vs. 0–3)	3.1	0.004	2.1	0.02
Dissected nodes (≥16 vs. <16)	1.0	0.91		
Margins (pos vs. neg)	2.7	0.15		
Multifocality (yes vs. no)	2.2	0.15		
LVI (yes vs. no)	1.7	0.30		
Luminal (other vs. A, B)	3.5	<0.0001	3.4	<0.0001
Age (≤50 vs. >50)	0.6	0.12		
RT-scheme (A vs. B)	1.2	0.53		
Amifostine (yes vs. no)	1.1	0.76		

## Data Availability

All data are available in the files of the Department of Radiotherapy and Oncology, Democritus University of Thrace. The data presented in this study are available on request from the corresponding author. The data are not publicly available due to ethical reasons.

## Appendix A

The λ-value reflecting the radiation dose lost (for treatment protraction) or gained (for treatment acceleration) daily is of critical importance to estimate the equivalence of accelerated to conventional schemes, in terms of antitumor efficacy and normal tissue toxicity.

Although such a value has not been directly reported for normal breast tissues, the λ-value can be postulated by applying simple radiobiological analysis (using the time-corrected EQD2 formula shown in the methods) on relevant results obtained from clinical trials, as follows:

- The Canadian trial [19] found equivalent breast toxicity between an hypoAR schedule of 42.5 Gy delivered in 16 fractions of 2.66 Gy within 22 days and a CRT scheme of 50 Gy delivering 25 fractions of 2 Gy within 33 days. Applying the time-corrected EQD2 formula for α/β = 4 Gy, the calculated λ-value is 0.26 Gy/day.
- The START-B trial [25] found less breast toxicity produced by an hypoAR schedule of 40 Gy delivered in 15 fractions within 19 days compared to the CRT scheme of 50 Gy delivering 25 fractions of 2 Gy within 33 days. This implies a λ-value of lower than 0.4 Gy.
- Assuming the equivalence between the FAST (5.7 Gy/week, 28.5 Gy in 29 days) vs. FAST-FORWARD (5.2 Gy/day, 26 Gy in 5 consecutive days) trials in terms of breast toxicity [18,30], the EQD2 with the time-correction formula provides a λ-value equal to 0.25 Gy/day (for a breast α/β = 4 Gy).
